# Globalisation, health and foreign policy: emerging linkages and interests

**DOI:** 10.1186/1744-8603-1-12

**Published:** 2005-07-29

**Authors:** John Wyn Owen, Olivia Roberts

**Affiliations:** 1 Former Secretary, The Nuffield Trust, London, UK; 2 Policy Officer, The Nuffield Trust, London, UK

## Abstract

A discussion of the growing links between the issues of globalisation, health and foreign policy. This article examines the effect this has on health, development and foreign policy communities in the UK and internationally and considers what steps the policy community must take to address the challenges and opportunities of this new relationship.

## Introduction

Professor David Fidler in his Maloy Lecture on 5th October 2004 at Georgetown University said, "The nature and extent of foreign policy attention devoted to health today is historically unprecedented" [1]. In his lecture he examined the nature of this increased attention, specifically whether "this political revolution reflects a transformation of foreign policy for the benefit of health, or a transformation of health for the benefit of foreign policy". He concluded that health does not transform how we think about foreign policy; rather foreign policy can transform how we think of health. In this article we examine why this is so, the effect this has on health, and what steps the policy community must take to address the challenges and opportunities of this new relationship.

Globalisation, health and foreign policy are themes that the Nuffield Trust has been analysing for a number of years, in association with its international partners. Globalisation can, at its core, be defined as a process of change affecting the nature of human interaction as boundaries become eroded across a range of spheres and along three dimensions: spatial, temporal and cognitive [2]. The rationale for engaging on this issue is based on three main developments.

• There are growing links between health policy and security and foreign policy, with developments in these fields having many implications for health, both in the United Kingdom and globally.

• Secondly, increasing globalisation has blurred the boundaries between domestic and foreign agendas, and the way we think and act in relation to health policy must adapt accordingly.

• Finally, links between health, foreign policy and security policy and development are increasingly been made, reinforcing the need to fully appreciate the place of health in the policy agenda.

Health has always been an issue in foreign policy but as its prominence increases, it is important to assess whether it is appropriately prioritised and how the government interacts with business and civil society on a national, regional (e.g. EU) and global basis. The HIV/AIDS pandemic, SARS, efforts to improve preparedness for bioterrorism, and the Framework Convention on Tobacco Control all provide recent examples where health concerns mix with high politics. SARS highlighted the necessity of global co-ordination of efforts to control communicable disease and the importance of urgent review and effective reform of the system of international health regulations. Since the events of 11th September 2001 the health and development agenda has also been widely linked with the foreign policy priorities of improving global security and preventing state failure. Bioterrorism has formed a prominent part of the health and security agenda. These examples illustrate how health has become a foreign policy and security issue for a range of actors both inside and outside government: there has been recognition that new and existing problems necessitate new responses and a scaling up of efforts. As a result, policy-makers in the previously rather distant fields of health, security and foreign policy must consider each other's work as they are confronted by the interplay of issues at the global level.

However, there are considerable differences between countries in their attitudes to the relationship between health and foreign policy. The 2002 Canadian Commission on future health care stated that access to health care is not only a domestic policy priority but also a key objective for foreign policy as well, and the promotion of human rights, including the right to health, is a fundamental principle of Canadian foreign policy [3]. In contrast, US foreign policy is shaped by the domestic political agenda with schemes such as PEPFAR heavily influenced by the present administration's value judgements, and less likely to be part of a co-ordinated approach with other countries [4]. The conditionalities that US foreign policy attitudes place on global health policy, such as the dominance of abstinence-based programmes in US AIDS funding [5], can restrict global health solutions and ultimately undermine the achievement of global health equity.

## Health and Security

The current inclusion in policy discussions of issues beyond national borders highlights the increasing debate about the implications of globalisation for health. While globalisation has perhaps become an over-familiar term it encompasses issues of enduring and profound significance: the opening of economies, increasing flows across borders, and increasing interdependence between people and places. As a result the distinction between domestic and foreign spheres is becoming more blurred. Inge Kaul, Director of Studies for the UNDP programme on global public goods, noted that the increase of problems in the global arena requires a more global perspective on foreign affairs in national policies and within those departments dealing with foreign affairs [6]. As Peter Hain, a UK Government Minister, wrote in his book 'The End of Foreign Policy', 'there is no longer such a place as abroad' [7].

These developments have many implications for health, presenting both risks and opportunities. Whilst states have sought to retain sovereignty over health care and health policy, the determinants of health often lies with global forces. The challenge is to maximise the benefits and minimise the harm of globalisation, keeping the questions of human rights and equity firmly in view. On the one hand states are forced to co-operate to solve their problems: this applies to health, peace, the environment and knowledge. On the other hand there is a trend towards subsidiarity or the principle of devolving power of decision-making to the lowest possible levels. Many areas of public policy that were considered to be national now spill across borders and are global in reach and impact. Foreign policy makers must therefore broaden their horizons when devising policies aimed at national interests.

Inevitably, when thinking of foreign policy, the issue of security and the protection of national interests are considered. Crucial policy goals, such as human security, are being reshaped in light of this global influence and issues such as health play a central part in making globalisation work. It is also an area of shared mutual concern that offers an opportunity to address some of the world's pressing problems. Providing health services and responding to health crises in regions experiencing or emerging from conflict is one of the most difficult challenges faced by national health systems, international organisations and humanitarian agencies. Conflict adds to the burdens faced by the health systems in many countries, creating additional need and diverting resources from other health priorities. Improved health systems can play a role in nation-building and reinforcing democratic principles. Within these contexts, health can be viewed as a core goal of socio-economic development efforts. A human security approach to health development offers a broadened and, arguably, more meaningful conception of security itself. However, there is a risk that health development may become linked to a narrower security agenda, which has a traditional focus on the national interests of the powerful, and on military intervention. It is therefore important that if healthcare is to act as a 'bridge to peace', as recognised by the WHO in their 2000 'Peace through health' initiative, the aims of one policy area are not sacrificed for the benefit of another [8].

## Health and Trade

Global trade rules, and in particular Trade-Related Aspects of Intellectual Property Rights (TRIPS), which form a central element of economic globalisation, have profound implications for health. There are five multilateral agreements on trade under the World Trade Organisation that are relevant for health. The General Agreement on Tariffs and Trade (GATT) allows countries under certain conditions to ban the import of products if necessary to protect public health. However, the protection of patent ownership within this Agreement can promote intellectual property above public health. The Agreement on the Application of Sanitary and Phytosanitary Measures (SPS) affects national policies for food safety, and runs the risk health and safety regulations being used as an excuse for protecting domestic producers. The Agreement on Technical Barriers to Trade (TBT) may have implications for the adoption of health and safety regulations, if they add to production costs. The General Agreement on Trade in Services (GATS) extends the concept of cross-border trade to include, for example investments in the hospital sector, thus potentially opening up health systems to privatisation. Finally, the TRIPS Agreement, though intended to strengthen the incentives to create new knowledge, made patented drugs more expensive and restricted the ability of poorer countries to obtain medicines by prohibiting access to the cheaper generic drugs. This was despite containing a measure to allow countries to manufacture drugs locally under conditions of a public health emergency. The complexity of these rules means that to ensure the protection and promotion of health interests, countries need to combine considerable expertise in economics and law, whilst interacting with other nations and multinationals in ways that they may not be experienced. Where countries have side-stepped the trade agreements in the interests of public health, the outcome has been legal reprisals by patent owners through the conduit of international agreements [9]. As stated in the 2002 Commission on the Future of Health in Canada, there is profound concern about the potential for trade agreements to unduly constrain future policy options and it is important for WTO members to ensure that efforts to liberalize trade do not override social policy objectives such as global health equity [10].

While aid funding and technical expertise are essential, professional and community links are also vital to support global health – policies for global health cannot be determined by governments alone. An assessment of Australia's development programme in 2002 concluded that foreign investment is four times greater than direct aid, and is more likely to have a major impact on health [11]. This highlights a need for cross-sectoral dialogue and co-operation in both developed and developing countries, which is one of the defining features of health as a foreign policy issue. The prospects for global health, the health of the poorest in particular, are bound up with the need for reform of trade tariffs and domestic subsidies, debt relief and aid flows. This is not a one-way relationship, however, or purely a developing world phenomenon. The disrupting effects of health problems, particularly acute infectious diseases, on trade and the economic and social repercussions are well-known, and were recently demonstrated in the SARS outbreak [12].

Given this relationship, we need a better understanding of globalisation in order to make it work better for health. Globalisation badly managed could contribute to worsening health and health inequalities, if a substantial number of people are marginalised and disadvantaged by the process. On the other hand we could make globalisation work to the advantage of all, rich or poor. This is a hard challenge but a moral imperative of the first order, as the Prime Minister of the United Kingdom stated in the DFID White Paper on *Making Globalisation Work to Eliminate World Poverty *[13].

## Health and Development

Health has long been recognised as a central feature of development and was acknowledged as an important element of preserving international peace and security after World War 2. The Commission on Human Security reported that deterioration in health in large parts of the developing world has occurred at a time of major advances in medical research and development in richer countries, particularly in epidemiology and basic biomedical sciences [14]. And as a recent report by UNAIDS shows, the challenge for health and development also lies in shaping the political, economic and social order [15].

The closely related problems of poverty and ill-health have been the subject of a number of global initiatives since the late 1990s. Many governments have signed up to the UN Millennium Development Goals but not all have incorporated them into their policy frameworks. Pledges on funding for health have been made at summits of the G8 countries, initiatives such as Global Fund and the new partnership of African development have generated some resources and the US has pledged additional money. However, further endeavour is needed to secure consensus, coordinate efforts, and practically deliver on commitments and to consolidate health as an enduring international priority.

This is still one of the main challenges in global health: moving beyond welcome but insufficient increases in dedicated resources towards clear commitments aimed at solving the problems we face. There are also dangers in associating health with narrow foreign policy goals and conditionalities that may in fact undermine health, such as user fees on primary health care and education. What is needed is a broad and integrated view of health and its determinants, its linkage to foreign policy, and support and opportunities for countries to articulate both their own needs and agree common positions. If this agenda becomes a top-down enterprise formulated by G8 countries and then sold to the rest of the world, it is likely to fail. SARS has shown the limits of current approaches to health challenges and lessons must still be learnt and re-learnt in order to deal with the threatened AIDS epidemics in Russia, China and India. SARS served to clearly demonstrate the key requirements of robust health policy that are required to enable a state to be 'resilient' to health challenges. These are: ability to assess potential health challenges; prevention as part of the policy mind-set; preparation; capacity to respond; and ability to rapidly recover. To succeed also requires the involvement and consent of empowered civil society. The problem is global and international but much of the solution must be local and social. The promotion of global health can be a positive form of engagement within the global community due to health's status as a 'global public good', a universal right for all. By focusing on global equity, policies can be viewed as alleviating some of the negative aspects of globalisation and the promotion of health and robust health systems can make a crucial contribution to both human development and global security. Health improvement or, in some countries, the prevention of further decline, is not only important for humanitarian reasons but is essential for social and economic development.

There are also clear and close correlations between poverty, environmental hazards and ill health. A key issue is urbanisation, with the world becoming more urban, specifically in developing countries. The growth in many mega-cities is not well regulated and is environmentally hazardous, with huge gaps in social services and infrastructure. The United Nations has recently scaled up its habitat programme and under the US Administration, USAid brought some focus to urbanisation but the effort is still under-resourced. The distinction between states and the cities within those states is crucial: foreign policy traditionally looked at the world as composed of states but it is now urban centres that connect the world, through the process of globalisation. To understand global health challenges we must keep in view the world of cities and the importance of urban public health.

## Governance

Global governance for health continues to evolve and become more complex and challenging, involving multiple actors and changing ideas of sovereignty. There is a need for integrated thinking, bringing health together effectively with other policy areas in the context of globalisation. We must continue to ask how appropriate current global, regional and sub-regional arrangements are for dealing with new and existing global challenges. In September 2003 Kofi Annan, Secretary-General of the United Nations, said, "We have come to a fork in the road. This may be a moment no less decisive than 1945 when the United Nations was founded" [16]. The UN was a divided organisation, with countries disagreeing about the war in Iraq and how best to respond to threats to their collective future, varying from weapons of mass destruction to HIV/AIDS and global warming. Left unattended, these problems reinforce each other. Disregard for a failing state today may contribute to the emergence of bastion of terrorism tomorrow. Afghanistan showed us that economic issues and health could undermine our ability to respond to conflict, with Afghanistan ranking at or near the bottom of every socio-economic indicator used to measure human and economic progress [17]. 23 years of conflict and the economic degradation associated with conflict decimated infrastructure, causing the decline of preventive healthcare, the unavailability of treatment facilities and drugs, and the lack of appropriately trained health care personnel.

Nowhere more so than in Africa does governance and health form a unique challenge. It is predicted that in ten years' time HIV/AIDS will have reduced significantly the capability of the South African Defence Force, undermining its ability to be an effective peacekeeper in the region [18]. This is one illustration of why a mature discussion is needed on globalisation, health and foreign policy matters, including the promotion of resilience, capability and leadership and co-ordination on global public health policy. A reformed United Nations is crucial to delivering this goal. Firstly, reform of the UN should aim not only to prevent conflict but when conflict has ended, to take action to develop institutions and build an enduring peace. Secondly, there is a role for the UN in developing agreements which provide necessary resources to efficiently achieve the Millennium Development Goals, focusing on what countries need to tackle poverty and to create better health care systems. Finally, there is a need for stronger international regimes to counter today's threats of poverty, disease, climate change and provide greater security from terrorism and weapons of mass destruction via tighter nuclear, biological and chemical controls and reinforced co-operation on counter-terrorism.

The political will of countries will be crucial and the health communities can contribute to raising awareness and engaging with the policy community to promote action. As medical students wrote in *The Lancet *on 3 November 2001:

For many the true meaning of globalisation hit home acutely after the terrorist attacks in the United States on 11th September. The health sector too is profoundly affected by changing global processes, from the horror of the HIV/AIDS pandemic to the increasing rates of refugees and migrants: from the controversy over global pharmaceutical patents to the health implications of the World Trade Organisation. The issues of the day all affect the work of a doctor. It is not no longer enough for medical curricula to teach about national medicine: our new doctors want and need more" [19].

In the case of the European Union, one suggested solution is an EU Global Health Strategy. At national or regional levels, the development of 'pathfinders' through research and consultation processes could provide a framework for future global health strategies. Such documents would encompass key elements and messages, incorporating important trends and issues in global health and taking a systematic and focused perspective on a nation's capabilities in global health. It could also highlight key principles policy formulation, specifically the ability to consider and appropriately balance the differing objectives and mechanisms within foreign affairs, trade, health, finance and home affairs.

In summary, the challenge is how to make globalisation work for health and to use health to foster better forms of globalisation. Implicit in the idea of making globalisation work is the contention that it is not working at the moment. Some may argue that this is not the case: global life expectancy continues to rise, the global economy expands, and scientific innovation and discovery proceed at seemingly exponential rates, unlocking the keys to increased health, wealth and happiness. However, we are aware, as never before, of the downside of our increasing interconnectedness and while large parts of the globe experience the positive story of globalisation, millions are cut off from it. Less than 10% of health research is directed towards the major health problems that affect 90% of the world's population. These failings must be addressed, not just for reasons of common humanity but for the fundamental reason that the negative externalities of economic globalisation may in time threaten its very foundations. Health has a central role to play in meeting the challenge of making globalisation work. The danger is that we will have health as a private good, health as exclusive and hierarchical, health as the preserve of only the rich, and health as a matter only of national security. This challenge is not just about technology, neither is it just about supply and demand, getting markets right, although both will play a role. The Nuffield Trust recognises the importance of extending the appreciation of health issues amongst policy-makers, and has been working on initiatives to bring together diverse members of policy to discuss global challenges. However, it is not just a matter for politicians, although they must of course play their part. This is fundamentally a challenge to our ability to act together at all levels that affect and are affected by these issues: the places we live; political communities and nations; across different countries; and in institutions of global governance. By mobilising the key actors we can begin to fulfil the promise of 'health as a bridge for peace' and reap the global benefits that this will bring.
